# Higher throughput assays for understanding the pathogenicity of variants of unknown significance (VUS) in the RPE65 gene

**DOI:** 10.1101/2025.01.31.635952

**Published:** 2025-02-05

**Authors:** Leila Azizzadeh Pormehr, Kannan Vrindavan Manian, Ha Eun Cho, Jason Comander

**Affiliations:** 1 Ocular Genomics Institute, Berman-Gund Laboratory for the Study of Retinal Degenerations, Mass Eye and Ear, Harvard Medical School, Boston, Massachusetts, USA

**Keywords:** RPE65, variant pathogenicity, variant of unknown significance (VUS), High-throughput assays, inherited retinal diseases, next-generation sequencing (NGS), retinitis pigmentosa, fluorescence activated cell sorting (FACS), voretigene neparvovec

## Abstract

**Purpose:**

*RPE65* is a key enzyme in the visual cycle that regenerates 11-cis retinal. Mutations in *RPE65* cause a retinal dystrophy that is treatable with an FDA-approved gene therapy. Variants of unknown significance (VUS) on genetic testing can prevent patients from obtaining a firm genetic diagnosis and accessing gene therapy. Since most *RPE65* mutations have a low protein expression level, this study developed and validated multiple methods for assessing the expression level of *RPE65* variants. This functional evidence is expected to aid in reclassifying *RPE65* VUS as pathogenic, which in turn can broaden the application of gene therapy for *RPE65* patients.

**Methods:**

30 different variants of *RPE65* (12 pathogenic, 13 VUS, 5 benign) were cloned into lentiviral expression vectors. Protein expression levels were measured after transient transfection or in stable cell lines, using Western blots and immunostaining with flow cytometry. Then, a pooled, high throughput, fluorescence-activated cell sorting (FACS) assay with an NGS-based sequencing readout was used to assay pools of *RPE65* variants.

**Results:**

There was a high correlation between protein levels measured by Western blot, flow cytometry, and the pooled FACS assay. Using these assays, we confirm and extend *RPE65* variant data, including that Pro111Ser has a low, pathogenic expression level. There was a high correlation between RPE65 expression and previously reported enzyme activity levels; further development of a high throughput enzymatic activity assay would complement this expression data.

**Conclusion:**

This scalable approach can be used to solve patient pedigrees with VUS in *RPE65*, facilitating treatment and providing *RPE65* structure-function information.

## Introduction

One of the primary goals of human genetics is to understand how genetic variation affects the function of genes and contributes to disease development.^1^ In fact, of more than 4 million missense identified variants, only about 2% have been definitively classified as pathogenic or benign. Most missense variants are classified as VUS.^2^ The current study is motivated by the observation that genetic testing for inherited retinal diseases (IRDs) gives ambiguous or negative results about one third of the time, often due to VUS.^3–7^ Proper pathogenicity classification of new or rare variants is important for a conclusive molecular diagnosis and the medical management of patients.^8^ This problem can have clinical consequences, preventing familial risk assessment, family planning, and access to an approved gene therapy or other investigational gene-specific therapies. In IRDs, this problem of VUS is particularly impactful for the *RPE65* gene, which was the first gene to have a corresponding FDA-approved gene therapy. Indeed, genetic testing results containing a *RPE65* VUS can prevent patients from obtaining treatment with an FDA-approved gene therapy, Voretigene neparvovec (Luxturna, Spark Therapeutics, Philadelphia, PA).^9,10^ Mutations in the *RPE65* gene account for 0.6–6% of RP and 3–16% of LCA/EORD cases,^11^ and patients are eligible for treatment only if they have documented biallelic pathogenic or likely pathogenic mutations.^12^

While there are a number of computational tools that can be used to predict pathogenicity^2,13,14^, they are not highly accurate.^15,16^ Even with advances in computational algorithms^17–19^, the level of accuracy may not be high enough when making medical decisions, including the decision to expose a patient to a gene therapy surgery specific to a certain genetic cause of disease. Additionally, ACMG guidelines do not allow a definitive diagnosis based on computational predictions alone.^20^ As a result, the use of validated, laboratory-based functional assays is considered strong evidence towards the reclassification of VUS into pathogenic variants.^2,6,7,13–20^

While the traditional method of investigating variant pathogenicity is to test one variant at a time, the benefits of producing this information at scale have resulted in the field of “functional genomics” in which parallelized, higher-throughput assays are used to assay pools of variants. Widespread use of functional genomics could improve the accuracy of variant interpretation in genes with both known and unknown associations with disease, generate information and reagents needed to test therapeutic agents, and inform the development of analytical tools for predicting variant pathogenicity. Therefore, the purpose of this study is to develop and validate higher-throughput expression assays for *RPE65* to provide expression information on a panel of *RPE65* variants at a higher accuracy than can be achieved by bioinformatic estimates alone.

Mechanistically, *RPE65* plays the central role in the retinoid cycle^21–23^ and encodes retinoid isomerohydrolase. The retinoid cycle enzymatic pathway allows continuous vision by regeneration of 11-cis from all-trans-retinal, which becomes part of the main chromophore of phototransduction in photoreceptor cells.^24–26^ More than 230 missense mutations lacking a clear pathogenicity classification or classified as VUS have been reported in public databases for *RPE65* (ClinVar). Many of these mutations introduce missense and nonsense mutations which affect the protein expression level or enzymatic activity, including via changing protein localization or stability.^27–29^ For example, missense mutation of G40S caused reduced catalytic activity to 2% of wildtype levels, and reduced protein levels to less than 40% of wild type levels.^23,30,31^

A traditional method for quantifying protein levels in cells is the Western blot, which is semi-quantitative when calibrated, but is low-throughput and has a limited dynamic range. More scalable quantification techniques can include use of fluorescent protein fusions (VAMP-seq)^32^, flow cytometry^33^, split luciferase systems^34^, and barcoded transcriptional reporters.^35^ Flow cytometry is particularly suited to higher-throughput assays implemented as pooled assays, as individual cells from a library can be rapidly separated for analysis. Fluorescence from immunostaining or from engineered fluorescent proteins can be used to sort cells based on the expression of cell surface and/or intracellular proteins. By combining FACS and next-generation sequencing (NGS), it is possible to obtain a more comprehensive understanding of thousands of variants.^36^ While many functional genomics studies use tags to facilitate protein detection^37,38^, this study uses only the native to human *RPE65* protein sequence to reduce the uncertainty that genetically encoded tags could disrupt the fidelity of the assays in a manner that is difficult to detect.

We hypothesized that cross-validating different analytical techniques with different antibodies and across a range of *RPE65* variants would evaluate the specificity and reproducibility of the reagents and allow for the development of a higher throughput RPE65 protein expression assay based on flow cytometry.

## Methods

### Selection of *RPE65* variants for study:

30 different *RPE65* variants were selected spanning a variety of pathogenicity levels: 12 pathogenic, 13 VUS, 5 benign. (See Supplemental Table 1, with HGVS nomenclature.) Wildtype RPE65 was also included. Among the variants that underwent analysis by multiple assays (Table 1), the negative control mutants that are known to have low expression levels included: G40S, R515W, G104V, P25L, P363T, G244V, and R124*. Y368H has conflicting expression level data and is known to have low activity. H241L and Y239D were selected as known mutations in the active site region. C112Y was reported as homozygous in IRD pedigrees^39,40^, which this study will consider as likely pathogenic. The positive control variants were A434V and N321K, which are known to have normal^30^ or slightly decreased^23^ activity and have a high maximum allele frequency in humans: A434V: 7.7% in African/African Americans and N321K: 3.5% in South Asians (gnomAD database v4.1.0). K294T was a likely benign variant, with somewhat conflicting activity levels reported^23,30^. The variants N248S, V189I, T94A, T390I were selected from a panel of VUS that were detected in IRD patients, provided by Spark Therapeutics.

A second batch of variants were selected for testing by flow cytometry analysis only (Table 2). A434=, T385=, and E352= are synonymous variants hypothesized to behave as wildtype. T86N, S533T, T105N, P111S, and N135K was selected because, at the time of initiation of this project, the variants were identified as VUS by the RPE65 Variant Curation Expert Panel (Lori Sullivan; personal communication). G193S was selected as a VUS from the ClinVar database. L450V is a potential hypomorphic allele from our institution’s patient cohort. D477G is a known dominant pathogenic mutation which produces a distinct phenotype compared to recessive *RPE65* mutations^23^. H527R is a known mutation in the active site region.

### Plasmid construction:

The human *RPE65* cDNA (NM_000329.3, without untranslated regions) amplified and was cloned into the NheI and BamH1 site of pMT_025 lentiviral expression vector (Addgene,#158579).^41^ Mutagenesis was used to create the DNA changes as listed in Supplementary Table 1. NGS or Sanger sequencing was performed to verify the correct sequence in all plasmids.

### Transient transfection of variants:

30 different variants of *RPE65* were used for transfection in HEK293T cells (ATCC, Cat no. CRL-3216). For transfection, HEK293T cells were seeded in 6-well(C6) or 12-well(C12) plates at a density of 4.3×10^4^ cells/cm^2^. The cells were maintained in Dulbeccòs Modified Eagle Medium (DMEM) supplemented with 10% fetal bovine serum (Thermo Fisher, Cat no. SH3007103). At 70–80% confluency, 2.5 μg plasmids were transfected per C6 well using Lipofectamine 3000 transfection reagent (Thermo Fisher, Cat no. L3000008) according to the manufacturer’s instructions. Cells were assayed 48 hours post-transfection.

### Generation of stable cell lines for *RPE65* variants:

Lentiviruses were prepared by transfecting HEK293FT cells with psPAX2 (Addgene no. 12260), pMD2.G (Addgene no. 12259) and pMT_025 RPE65 variant using Lipofectamine LTX according to the manufacturer’s instructions. The viral supernatant was harvested, concentrated and transduced into HEK293T cells at MOI of <0.3. The transduced cells were selected using puromycin to generate stable lines. For additional details, see Supplementary Methods.

## Western blotting

Whole-cell lysates were prepared using RIPA buffer from the transient and stable cell lines. After quantification, the lysates were loaded on SDS-PAGE, followed by dry transfer using iBlot^™^ 2 system (Invitrogen, Carlsbad, CA). Blots were incubated with primary antibodies of *RPE65* and ß-actin overnight at 4°C followed by respective secondary antibodies. The blots were detected using the Odyssey CLx infrared imaging system (LICOR, Lincoln, Nebraska). LICOR Image Studio was used to quantify the band intensity, and the data was normalized to the expression level seen in the wild-type (WT) *RPE65* sample. For additional details, see Supplementary Methods.

### Quantification of RPE65 expression levels by flow cytometry:

Initial experiments used the PETLET *RPE65* antibody (not shown; from the Redmond laboratory), but the following experiments were conducted with commercially available antibodies that are more broadly available. Cells were collected for flow cytometry analysis 48 hours post transfection. For stable cells lines, cells were collected at 80% confluency. After washing with PBS, cells were collected by trypsinization, centrifuged at 1200 g for 4 minutes, and washed with PBS. The cells were resuspended and fixed in 4% paraformaldehyde (PFA, Electron Microscopy Sciences, Cat no. 15714) in PBS for 20 min and washed with PBS and kept at 4°C until staining. For staining of *RPE65*, cells were permeabilized with 0.02% saponin (Sigma, Cat no. 47036) in PBS for 12 minutes, followed by blocking in 3% BSA in PBS. After blocking, cells were incubated with primary antibodies described above at a final concentration of 1:1500 in blocking buffer for 1 hour. After washing with PBS, Alexa fluor-488 secondary antibodies goat anti-mouse IgG1 (Invitrogen, Cat no. A21121) and goat anti-rabbit IgG (Invitrogen, Cat no. A27034) were used at a final concentration of 1:500 in blocking buffer for 1 hour. After a PBS wash, cells were resuspended in appropriate volume of PBS and analyzed on a Miltenyi BioTec MACSQuant flow cytometer. Flow cytometry results were analyzed with FlowJo Software v10. The resulting data were averaged across multiple independent transfections (N= 3–5).

### Pooled quantification of *RPE65* expression levels by FACS and NGS:

Stable cell lines expressing RPE65 variants (N=16 variants) were pooled together, fixed using 4% PFA, and stained for FACS as described previously for flow cytometry. The stained cells were sorted on Sony SH800 or MA900 FACS machines by their *RPE65* expression level, where the “high” and “low” gates contained the top and bottom 18% of fluorescent intensity, respectively. The cells were lysed and the genomic DNA from *RPE65*^high^, *RPE65*^low^, and un-sorted cells was uncrosslinked (manuscript in preparation) and extracted. The open reading frame of the lentiviral integrands was amplified and quantified using next-generation sequencing and compared to flow cytometry expression results. Briefly, the open reading frame containing the *RPE65* variants was amplified using primers XY304 (ATTCTCCTTGGAATTTGCCCTTT) and XY305 (CATAGCGTAAAAGGAGCAACA). NGS libraries (170–280 bp) were created using fragmentation and sequenced on the Illumina MiSeq. Fastq files were aligned to a reference sequence containing the pMT025-RPE65 plasmid. Variants were quantified using bam-readcount^42^ and normalized to read depth. The relative expression level for each variant in the pool was calculated as the variant count in the high gate divided by the count in the low gate.^33^

## Results

### Assay development

#### Measuring the linearity of western blot detection of *RPE65* and Beta-actin

**Figure 1.**
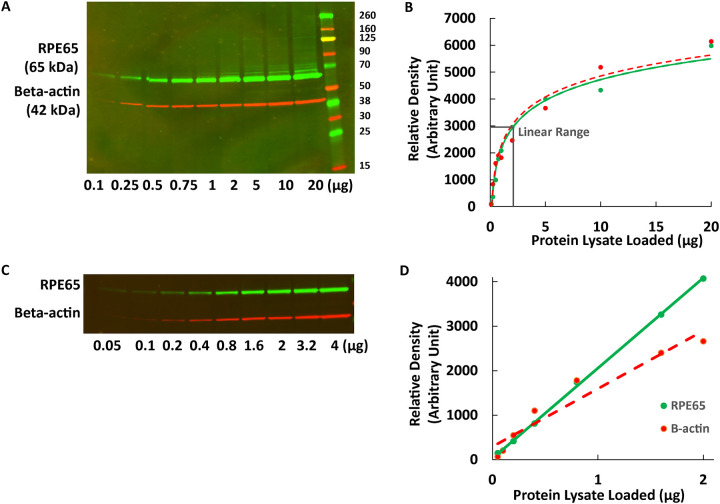
Linear range determination for RPE65 and ß-actin by Western blotting (A: 0.1 – 20 μg lysate, C: 0.05–4 μg lysate), with corresponding densitometry results (B,D). For D, the intensity was linearly correlated with protein loading amount for RPE65 (r=0.99) and for ß-actin (r=0.96).

**Figure 2.**
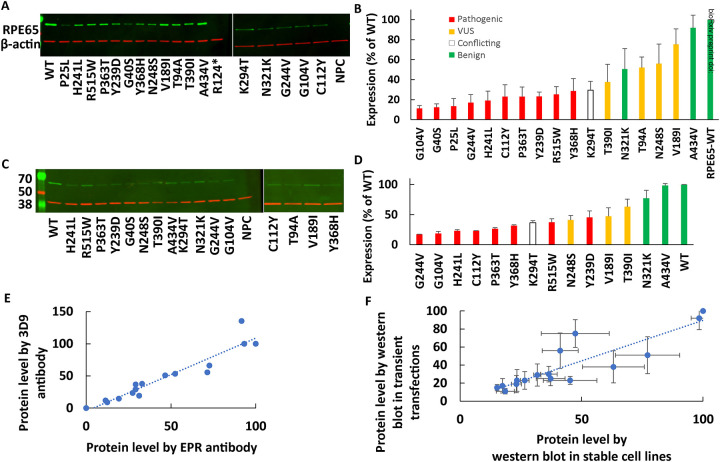
Protein expression levels of different RPE65 variants were assayed by Western blotting (A: transient transfections, C: stable cells lines) with corresponding densitometry results (B,D). In panels B and D, expression levels were normalized to ß-actin levels and are expressed as a percentage of wildtype levels (N=2–3), with known mutants shown in red, VUS in yellow, benign variants in green, and a variant with conflicting interpretations in white. E: A high correlation was observed between the expression levels measured using 2 different RPE65 antibodies (r=0.95, N=1). F: A high correlation was observed between the average measurement of RPE65 variants expressed by transient transfection and by stably-expressing cell lines (r=0.89, N=2).

To calibrate the detection range and linearity of *RPE65* and ß-actin via Western blotting, we prepared HEK293T cell lysates transiently transfected with *RPE65*-wildtype (WT) and loaded 0.1 −20 μg of whole cell lysates for immunoblotting (N=2). Densitometry showed that the linear quantification range was narrow and required using relatively small amounts of protein (0.25–2 μg total lysate) and a small amount of antibody (1:5000 dilution of anti-RPE65 EPR), as seen in Figure 1 A, B. There was no *RPE65* specific band (65 kDa) observed in untransfected cell lysate (not shown and also see Figure 2 below). To further delineate the proportional linear range, even smaller amounts of diluted protein lysate (0.05–4 μg) were used, which indicated that the proportional linear range for both *RPE65* and ß-actin (Santa Cruz) were 0.1–2 μg (Figure 1 C, D). For stable cell lines that contained only a single copy of the *RPE65* transgene, there was an approximately 4-fold lower expression in the stable lines compared to transient transfection, and protein lysate ranging from 1–20 μg was tested. Four μg was the higher end of linear range (not shown). For further analysis of the different variants by Western blot, 1 μg protein lysate for transient transfections and 4 μg protein lysate for stable cell lines was used per lane. Total protein staining (Revert 700) was not accurately detectable by Li-cor Odyssey densitometry using the small protein amounts required for antibody detection within the linear range (not shown), therefore ß-actin was used as a loading control for normalization. For the single-copy stable cell lines only, the use of 4 μg protein lysate was slightly beyond the linear range of ß-actin detection but was required for detection of *RPE65*. The use of 1 μg protein lysate from the transient transfections was within the linear detection range for both *RPE65* EPR and ß-actin. Similar results were obtained with the “3D9” *RPE65* antibody, but for Western blotting the linear range and detection limit were slightly lower (not shown). Altogether, this data indicated that the best combination of antibodies and conditions for the most accurate quantification of *RPE65* protein levels by Western blot was “EPR” (1:5000) and Santa Cruz sc-47778 anti-ß-actin (1:5000). (See Methods.) Therefore, these antibodies and concentrations were used for comparing *RPE65* variant expression levels using Western blotting below.

#### Measurement of the protein levels of different variants by Western blot in transient transfections and stable cell lines

To analyze the effects of different variants on protein level of *RPE65* different variants (benign positive controls, pathogenic negative controls, and VUS), Western blotting was used to quantify protein levels in lysates from transient transfections (Figure 2A and B) and stable cell lines (Figure 2C and D). Using either transient transfection or stable cell lines, all of the pathogenic variants showed <50% of the wildtype protein level. Figure 2E shows a high correlation between staining of different variants with 2 different anti-*RPE65* antibodies (r=0.95), giving evidence for the specificity of the detected antigens. Also, the results showed a high correlation between measured protein levels in transiently transfected cells and in stable cell lines (r=0.88, Figure 2F).

#### RPE65 protein levels of different variants using flow cytometry

**Figure 3.**
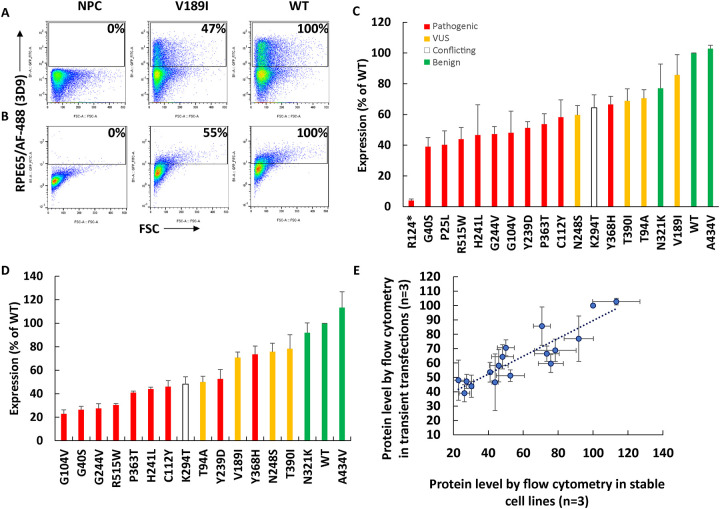
Measurement by flow cytometry of protein levels of different variants in transient transfections (A,C) and stable cell lines (B, D). Flow cytometry dot plots of untransfected cells (NPC), V189I, and wildtype RPE65 in transiently transfected cells (A) and stable cell lines (B). The transiently transfected cells express higher antigen levels than the stable cells lines with single integrations. Quantification of protein level of different variants (red: pathogenic, yellow: VUS, green: benign, white: conflicting) in transiently transfected cells (N=3). (C) and stable cell lines (D) (N=3). E: High correlation in the measurement of protein levels by flow cytometry between transiently transfected cells and stable cell lines (r=0.89, N=3).

Flow cytometry was optimized to measure the protein levels of different *RPE65* variants using transient transfections and stable cell lines. Although the EPR antibody was best for Western blotting, for flow cytometry, antibody 3D9 showed a wider dynamic range than EPR for measuring the protein level across different variants (not shown). 3D9 was used for further staining for flow cytometry (Figure 3). Flow cytometry results using transiently transfected cells and stable cell lines were highly correlated (Figure 3E) (r=0.89, N=3).

#### Correlations between assays

**Figure 4.**
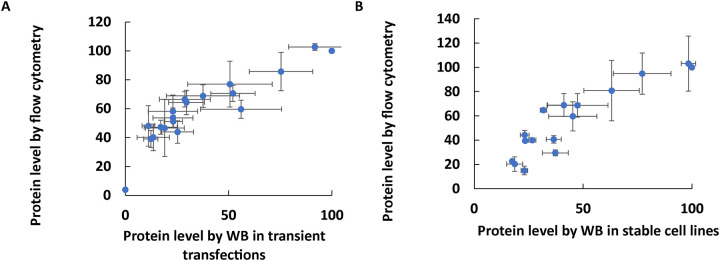
Optimized measurement of RPE65 variant protein expression levels using Western blotting with EPR antibody compared to flow cytometry with 3D9 antibody. A high correlation was obtained in both transiently transfected cells in panel A (r=0.91, N=3) and stable cell lines in panel B (r=0.91, N=2).

**Figure 5.**
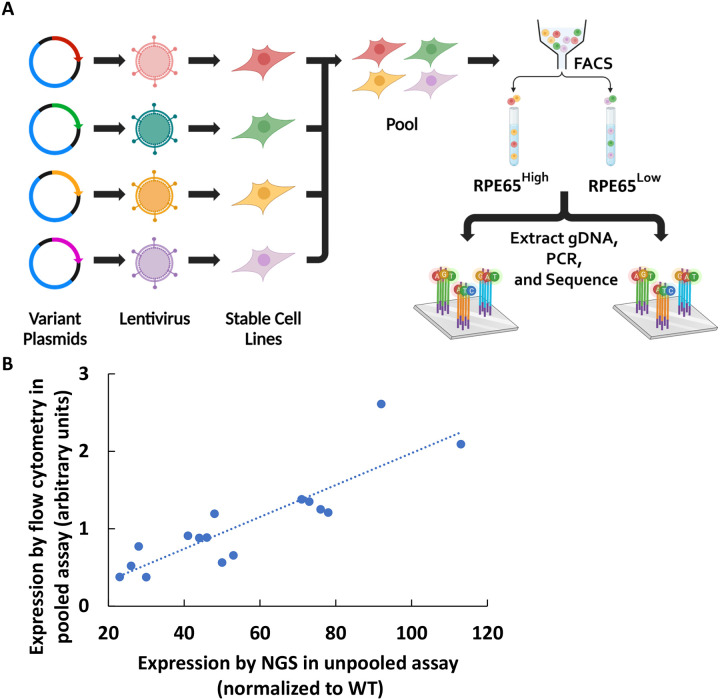
A: Workflow of pooled library assay, B: Comparing the measurement of expression levels in pooled library by NGS and flow cytometry (unpooled). There is a high correlation between pooled assay and un pooled flow cytometry assay (r=0.87, N=3)

Using Western blotting for quantifying protein levels in cells is not easily scalable for measuring large numbers of different variant protein expression levels. A flow cytometry assay was optimized for measuring different variants and the method showed high correlation to values measured by Western blotting using transiently transfected cells (Figure 4A, r=0.91) and stable cell lines (Figure 4B, r=0.91). Next, a pooled assay was developed for a higher throughput flow cytometry assay based on pooled RPE65 stable cell lines (Figure 5A). A strong correlation was observed in the RPE65 variant expression levels between the unpooled assay and the higher-throughput pooled assay (Figure 5B) (r=0.87).

**Figure 6.**
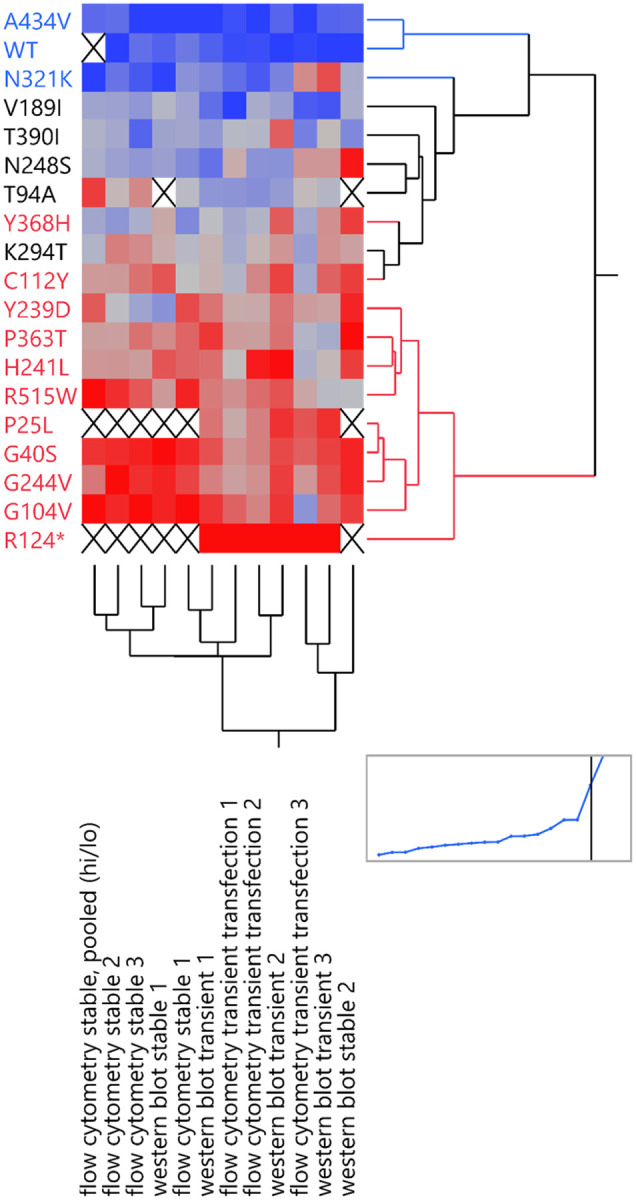
A heatmap comparing assay results across different assays (columns) and different RPE65 variants (rows). A hierarchical clustering algorithm was used to group together similar data and produce row and column dendrograms. Blue denotes high expression and red denotes low expression. An X indicates missing data.

In summary, the five assays tested above showed good agreement, as shown graphically in Figure 6, validating and giving support to the specificity and dynamic ranges of the assays tested above. The rare outlier points are also discussed below.

#### Additional variants tested

**Figure 7:**
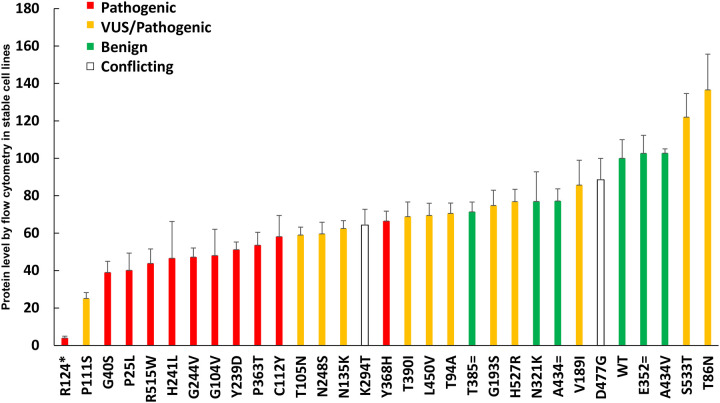
Protein levels of different RPE65 variants, measured by flow cytometry after transient transfection, N=3 (red: pathogenic, yellow: VUS, green: benign)

Next, based on the validation of the assays shown above, additional *RPE65* variants (see Table 2 and Methods) were assayed for protein expression levels using transient transfection, staining with the 3D9 antibody, and the unpooled flow cytometry readout. Figure 7 shows the expression levels of these additional variants. There was substantial but not complete separation between the values obtained by the positive controls (green) and the negative controls (red), as discussed below. P111S, initially a VUS, was identified as having pathogenic expression levels. Three positive control synonymous variants were hypothesized to have the same expression as the wildtype protein, but two had T385= and A434= had slightly decreased levels (71+/−5.23 % and 77+/−6.5 %, respectively), in the context of their expression from a cDNA without introns.

## Discussion

With the approval of *RPE65* gene therapy and the increased number of inherited retinal disease patients undergoing DNA sequencing, there is an increasing need for higher throughput pathogenicity assays for *RPE65* variants. The development of a higher throughput assay is often based on a simple but robust technique, and this study evaluated a straightforward protein detection assay to identify *RPE65* variants with pathogenic protein expression levels.

### Comparing the protein level measured in this study with the other studies

By one estimate, 80% of mutations are misfolding, 10% are active site mutations, and 10% have another mechanism.^43^ Of course, these proportions and categories can vary by gene, and remain to be fully defined for *RPE65*. Based on current understanding, the different mechanisms that can be involved in pathogenicity of different *RPE65* variants include: misfolding, loss of catalytic activity, toxic gain of function, mis-localization, or aggregation of the mutated protein.

Regarding variants that are thought to be at or near the active site, we analyzed the expression level of 3 active site variants – H241L, H527R and Y239D. The mechanism of pathogenicity in the active site is different than elsewhere^31^, but interestingly our study showed that the protein levels of H241L and Y239D are also low. In fact every pathogenic variant tested by Western blot showed an expression level of 29% of wildtype or lower. The missense H527R variant was originally reported as a VUS in ClinVar and observed in individual(s) with clinical features of *RPE65*-related conditions, but has since been reclassified as pathogenic.^44^ *RPE65* with H527R mutation did not show any catalytic activity.^31^ This study show a slightly decreased expression level (77%, Figure 7). Another variant close to active site cavity, Y239D, showed a pathogenic expression level by Western blot and flow cytometry (Table 1). However, most disease-associated missense mutations in *RPE65* are non-active site mutations.^45^

Regarding the dominant variant D477G, this variant showed wildtype expression levels in HEK293T cells, consistent with a past study showing normal expression, localization, and catalytic activity in NIH3T3 cells.^46^ More recent studies have showed a decrease of expression levels in a knock-in mouse model^47^ and retinal degeneration only when exposed to environmental light.^48^

Regarding variants that are thought to cause misfolding and rapid degeneration of misfolded protein, including G40S, R515W, G104V and G244V, showed significantly decreased protein levels compared to wildtype (less than or equal to 29% of wildtype by Western blot).^27,28,30,49,50^ Also, mouse models of the P25L and R91W mutations showed decreased protein levels of the mutants.^51,52^ This decrease in protein level is likely biologically relevant; the catalytic rate of *RPE65* is so low that high expression of the protein plays the compensatory role for its weak enzymatic activity.^21,23^ The high stability of *RPE65* with half-life >10 hours and low degradation rate lead to high abundance in the retinal pigment epithelium.^53^

Regarding variants that may be benign, K294T was initially shown to have a severely decreased activity level.^23^ However, as seen in dbSNP (rs61752901; accessed 12/25) a high allele fraction in Latino populations of 1.1–3.1% indicates that it is benign. The K294T variant was reported in the heterozygous state in LCA patients.^30^ Unfortunately, both a later study^30^ and this study found borderline expression or activity levels. While we believe this variant is benign, the borderline levels do not allow that to be demonstrated definitively on a biochemical level and cannot rule out that it is hypomorphic. The other two benign variants in this study, A434V and N321K, showed normal expression.

Regarding variants that are in the amphipathic α-helix membrane targeting motif (aa 107–125), this study evaluated C112Y and P111S, which both showed pathogenic expression levels, with P111S showing the lowest expression of any missense variant tested in this study (Figure 7). Residue C112 (studied as C112A by Uppal et al) plays an important role in palmitoylation and localization of the protein into the membrane.^54,55^

### Interpreting the meaning of RPE65 expression levels

The usual mechanism causing decreased protein levels of the pathogenic *RPE65* variants is proteasomal degradation of the misfolded protein.^31^ In this study and others, the relative tendency of a particular protein sequence to misfold should be proportional in heterologous cells compared to the disease target cell, although this was not formally tested in this study. The protein detection assay in this study was successfully scaled up to a pooled assay, which in future studies can be applied efficiently to a much larger number of *RPE65* variants. However, while the absence of protein expression is good evidence of a pathogenic mutation (and can provide immediately useful information in that case), a wildtype-like expression level does not guarantee that a variant is benign. Our laboratory has been exploring the development of a higher throughput *RPE65* activity assay, intended to detect “active site” or other mutations which would affect enzyme activity but not protein levels. Despite trying to include such mutations in the panel tested, Table 1 and Figure 6 show how the protein detection assay mirrors known activity levels surprisingly well. As the number of variants tested increases, however, it would be expected to find a small fraction of “false negative” results using a protein detection assay alone.

Table 1 summarizes the protein level of different variants in previous studies compared to the current study. Both Table 1 and Figure 6 show a broad agreement between both the literature values and the assays used in this study, and among the different antibodies and techniques used. This lends higher confidence to the assays’ specificity and accuracy. The most robust and sensitive assay was Western blotting using transient transfection, and the low expression levels of the stable cell lines were limited by the single copy transgene and the presumed sensitivity of the antibody. In the pooled flow cytometry assay, the quantitative nature of counting the cells in the high versus low sorting gates, all in the same tube, is likely to increase the internal consistency of the assay compared to unpooled assays performed on separately stained and analyzed samples. While thanks to these advantages the pooled assay gave good results (Figure 6), the preferred antibody for flow cytometry, 3D9, had a moderate brightness / staining index; the dynamic range of the assay could be improved by developing a brighter antibody or, less ideally, using an epitope tag.

While the assays in this study generally had broad agreement, one specific variants (T94A) showed some disagreement between assays (Figure 6). Specifically, T94A showed borderline levels in all unpooled assays but a low level in the pooled flow cytometry assay. Of note, the T94A stable cell line did not grow well, indicating a possible toxicity of transgene expression. Selection of unusual clones during the stable cell derivation could produce artifactual results, though this does not apply to transient transfections.

Regarding the determination of an expression level which should be considered pathogenic, the pathogenic mutants showed less than or equal to 30% of protein level compared to wild type by Western blot of transiently transfected cells (Table 1) and consistent with Western blot results from other studies.^30,53^ Though the flow cytometry and Western blot results show a very high correlation (Figure 4), the Western blot values are likely more accurate on an absolute scale when comparing to external studies. By flow cytometry of transiently transfected cells, the dynamic range of the assay was slightly compressed, and less than 60% of wild type expression was always pathogenic (Table 1 and Figure 7), with 60–70% in an intermediate zone, and >70% always benign. In summary, pathogenic could be considered <30% of wild type by Western blot and <60% of wildtype by flow cytometry after transient transfection, when using the protocols of this study. While these simple numerical cutoffs are easy to understand and apply, as is often the case, many such assays have an indeterminate region or “grey area”.^56^ Ideally, the probability of pathogenicity would be expressed as a quantitative (Bayesian) probability,^57^ but for *RPE65* this will require data across a larger number of variants. Adding data from an *RPE65* activity assay would be beneficial as well.

### Reclassifying variants of unknown significance

Using the above criteria, P111S is solidly in the pathogenic range of expression levels. T105N, N248S, and N135K have borderline expression levels that may be pathogenic. V189I, S533T, and T86N show wildtype expression levels, but without an activity assay, no strong conclusions can be made.

In conclusion, all pathogenic *RPE65* variants tested show a low protein level, and validated protein expression assays can be used to reclassify the pathogenicity of VUS.^58^ Activity and localization assays would additionally identify the rare variants that have normal expression levels but lack activity.

Future work may include using the pooled assay to assay hundreds or thousands of variants. This generation of functional data will aid in the diagnosis and treatment of patients with *RPE65*-associated retinal degeneration. Furthermore, developing formal rules for the use of this functional data for classifying the pathogenicity of variants is ongoing in the Leber congenital amaurosis / early onset retinal dystrophy Variant Curation Expert Panel (LCA/eoRD VCEP) sponsored by NIH/NIGMS.

## Supplementary Material

1

## Figures and Tables

**Table 1.** Comparing expression levels of different known RPE65 variants between the results in this study and in previous studies. Results are expressed as a percent of wildtype. The color code for values is green=100 and red =0. The color code for pathogenicity is green = benign (b), yellow = VUS (u), red=pathogenic (p). Asterisks denote the number of stars in the ClinVar evidence rating.

				Transient transfection		Stable cell lines					
				Western blot	Flow cytometry	Flow cytometry	Flow cytometry pooled	Western blot	Expression	Activity	References
Variants	Pathogenicity	ClinVar as of 9/18/24	Clinvar variant ID	This study		This study			Literature	Literature	
p.Arg124 *	Stop mutation			0	4						
p.Gly104 Val	Pathogenic	likely p*	98864	11	48	23	18	19	16.7	0	28
p.Gly40 Ser	Pathogenic	p***	98830	12	39	26	25	15	~35, ~20	<2, 1.65, 1.3	23,30,31
p.Pro25 Leu	Pathogenic, “hypomorph”	p***	437985	14	40				24.33	7.75	25
p.His241 Leu	Pathogenic	p	812407	19	47	44	42	23		Not Detected	31
p.Gly244 Val	Pathogenic	path according to Bereta et al		17	47	28	37	17	~20	0	49
p.Arg515 Trp	Pathogenic	p***	13120	25	44	30		37	~25	Very low	27
p.Pro363 Thr	Pathogenic, “hypomorph”	p***	13117	23	54	41	44	27	~30	0	29
p.Tyr239 Asp	Pathogenic	p***	98889	23	51	53		45	~20	4.59, 1.3	30,31
p.Cys112 Tyr	Pathogenic	likely p		23	58	46	42	23			39,40
p.Tyr368 His	Pathogenic	p***	29870	29	66	73		32	~20, ~100	0	50,53
p.Lys294 Thr	Conflicting	likely b***	92859	30	64	48		36	~100	16, 68	23,30
p.Thr390 Ile	VUS	u*	851559	38	69	78	58	63			
p.Thr94A la	VUS	u**	1045866	52	71	50	27				
p.Asn321 Lys	Benign	b***	92860	51	77	92		77	~130	127	30
p.Asn248 Ser	VUS	u*	960875	56	60	76		41			
p.Val189 Ile	VUS	u***	876133	75	86	71	66	47			
p.Ala434 Val	Benign	b***	98836	92	103	113	100	98	~100	55, 110	23,30
WT	Wild type	(b)		100	100	100		100	100	100	

**Table 2.** Expression levels of additional RPE65 variants that were assay using the transient transfection method. The color code for pathogenicity is green = benign (b), yellow = VUS (u), red=pathogenic (p). Asterisks denote the number of stars in the ClinVar evidence rating.

Variants	Original Pathogenicity	ClinVar pathogenicity as of 9/18/24	ClinVar variant ID	Transient transfection and flow cytometry in this study
p.Pro111Ser	VUS	p***	281715	25.1
p.Gly193Ser	VUS	u***	962032	74.8
p.Thr105Asn	VUS	p*	1521093	59.0
p.Asn135Lys	VUS	u*	1065765	62.5
p.Leu450Val	VUS			69.5
p.Thr385=	Benign	u*	2234932	71.4
p.His527Arg	VUS	p***	1445004	76.9
p.Ala434=	Benign			77.2
p.Asp477Gly	Pathogenic, dominant	p/likely p**	750796	88.5
p.Glu352=	Benign			102.7
p.Ser533Thr	VUS	u***	870342	122.0
p.Thr86Asn	VUS	u***	1026379	136.6

## References

[1] Chakravorty S. & Hegde M. Gene and variant annotation for Mendelian disorders in the era of advanced sequencing technologies. Annual review of genomics and human genetics 18, 229–256 (2017). 10.1146/annurev-genom-083115-022545 28415856

[2] Cheng J. Accurate proteome-wide missense variant effect prediction with AlphaMissense. Science, eadg7492 (2023). 10.1126/science.adg7492 37733863

[3] Laddach A., Ng J. C. F. & Fraternali F. Pathogenic missense protein variants affect different functional pathways and proteomic features than healthy population variants. PLoS Biology 19, e3001207 (2021). 33909605 10.1371/journal.pbio.3001207 PMC8110273

[4] Brandes N., Goldman G., Wang C. H., Ye C. J. & Ntranos V. Genome-wide prediction of disease variant effects with a deep protein language model. Nature Genetics, 1–11 (2023). 10.1038/s41588-023-01465-0 PMC10484790 37563329

[5] Brandes N., Weissbrod O. & Linial M. Open problems in human trait genetics. Genome Biology 23, 131 (2022). 35725481 10.1186/s13059-022-02697-9 PMC9208223

[6] Nash B. M., Wright D. C., Grigg J. R., Bennetts B. & Jamieson R. V. Retinal dystrophies, genomic applications in diagnosis and prospects for therapy. Translational pediatrics 4, 139 (2015). 26835369 10.3978/j.issn.2224-4336.2015.04.03 PMC4729094

[7] Stanwyck L. K., Place E. M., Comander J., Huckfeldt R. M. & Sobrin L. Predictive value of genetic testing for inherited retinal diseases in patients with suspected atypical autoimmune retinopathy. American Journal of Ophthalmology Case Reports 15, 100461 (2019). 31193260 10.1016/j.ajoc.2019.100461 PMC6523031

[8] Motta F. L. Pathogenicity reclassification of RPE65 missense variants related to leber congenital amaurosis and early-onset retinal dystrophy. Genes 11, 24 (2019). 31878136 10.3390/genes11010024 PMC7016655

[9] Maguire A. M., Bennett J., Aleman E. M., Leroy B. P. & Aleman T. S. Clinical perspective: treating RPE65-associated retinal dystrophy. Molecular Therapy 29, 442–463 (2021). 33278565 10.1016/j.ymthe.2020.11.029 PMC7854308

[10] Gao J., Hussain R. M. & Weng C. Y. Voretigene neparvovec in retinal diseases: a review of the current clinical evidence. Clinical Ophthalmology, 3855–3869 (2020). 33223822 10.2147/OPTH.S231804 PMC7671481

[11] Aoun M. Inherited retinal diseases due to RPE65 variants: from genetic diagnostic management to therapy. International Journal of Molecular Sciences 22, 7207 (2021). 34281261 10.3390/ijms22137207 PMC8268668

[12] Sodi A. RPE65-associated inherited retinal diseases: consensus recommendations for eligibility to gene therapy. Orphanet J Rare Dis 16, 257, doi:10.1186/s13023-021-01868-4 (2021). 34088339 PMC8176684

[13] Ng P. C. & Henikoff S. SIFT: Predicting amino acid changes that affect protein function. Nucleic acids research 31, 3812–3814 (2003). 12824425 10.1093/nar/gkg509 PMC168916

[14] Jumper J. Highly accurate protein structure prediction with AlphaFold. Nature 596, 583–589 (2021). 34265844 10.1038/s41586-021-03819-2 PMC8371605

[15] Raraigh K. S. Functional assays are essential for interpretation of missense variants associated with variable expressivity. The American Journal of Human Genetics 102, 1062–1077 (2018). 29805046 10.1016/j.ajhg.2018.04.003 PMC5992123

[16] Anderson C. L. How functional genomics can keep pace with VUS identification. Frontiers in Cardiovascular Medicine, 1711 (2022). 10.3389/fcvm.2022.900431 PMC9291992 35859585

[17] Ioannidis N. M. REVEL: an ensemble method for predicting the pathogenicity of rare missense variants. The American Journal of Human Genetics 99, 877–885 (2016). 27666373 10.1016/j.ajhg.2016.08.016 PMC5065685

[18] Adzhubei I., Jordan D. M. & Sunyaev S. R. Predicting functional effect of human missense mutations using PolyPhen-2. Current protocols in human genetics 76, 7.20. 21–27.20. 41 (2013). 10.1002/0471142905.hg0720s76 PMC4480630 23315928

[19] Rentzsch P., Witten D., Cooper G. M., Shendure J. & Kircher M. CADD: predicting the deleteriousness of variants throughout the human genome. Nucleic acids research 47, D886–D894 (2019). 30371827 10.1093/nar/gky1016 PMC6323892

[20] Richards S. Standards and guidelines for the interpretation of sequence variants: a joint consensus recommendation of the American College of Medical Genetics and Genomics and the Association for Molecular Pathology. Genetics in medicine 17, 405–423 (2015). 25741868 10.1038/gim.2015.30 PMC4544753

[21] Moiseyev G., Chen Y., Takahashi Y., Wu B. X. & Ma J.-x. RPE65 is the isomerohydrolase in the retinoid visual cycle. Proceedings of the National Academy of Sciences 102, 12413–12418 (2005). 10.1073/pnas.0503460102 PMC1194921 16116091

[22] Ma J. X., Chen Y., Takahashi Y. & Moiseyev G. RPE65 is the isomerohydrolase in the retinoid visual cycle. Investigative Ophthalmology & Visual Science 46, 1057–1057 (2005). 10.1073/pnas.0503460102 PMC1194921 16116091

[23] Redmond T. M. Mutation of key residues of RPE65 abolishes its enzymatic role as isomerohydrolase in the visual cycle. Proceedings of the National Academy of Sciences 102, 13658–13663 (2005). 10.1073/pnas.0504167102 PMC1224626 16150724

[24] Cai X., Conley S. M. & Naash M. I. RPE65: role in the visual cycle, human retinal disease, and gene therapy. Ophthalmic genetics 30, 57–62 (2009). 19373675 10.1080/13816810802626399 PMC2821785

[25] Lorenz B. A novel RPE65 hypomorph expands the clinical phenotype of RPE65 mutations. A comprehensive clinical and biochemical functional study. Investigative ophthalmology & visual science 49, 5235 (2008). 18599565 10.1167/iovs.07-1671 PMC5015590

[26] Nikolaeva O., Takahashi Y., Moiseyev G. & Ma J.-x. Negative charge of the glutamic acid 417 residue is crucial for isomerohydrolase activity of RPE65. Biochemical and biophysical research communications 391, 1757–1761 (2010). 20043869 10.1016/j.bbrc.2009.12.149 PMC2812700

[27] Li S. Temperature-sensitive retinoid isomerase activity of RPE65 mutants associated with Leber Congenital Amaurosis. The journal of biochemistry 158, 115–125 (2015). 25752820 10.1093/jb/mvv028 PMC4516983

[28] Yang U. Utility of in vitro mutagenesis of RPE65 protein for verification of mutational pathogenicity before gene therapy. JAMA ophthalmology 137, 1381–1388 (2019). 31580392 10.1001/jamaophthalmol.2019.3914 PMC6777234

[29] Chen Y., Moiseyev G., Takahashi Y. & Ma J.-x. Impacts of two point mutations of RPE65 from Leber’s congenital amaurosis on the stability, subcellular localization and isomerohydrolase activity of RPE65. FEBS letters 580, 4200–4204 (2006). 16828753 10.1016/j.febslet.2006.06.078

[30] Philp A. Predicting the pathogenicity of RPE65 mutations. Human mutation 30, 1183–1188 (2009). 19431183 10.1002/humu.21033 PMC2717180

[31] Li S. Rescue of enzymatic function for disease-associated RPE65 proteins containing various missense mutations in non-active sites. Journal of Biological Chemistry 289, 18943–18956 (2014). 24849605 10.1074/jbc.M114.552117 PMC4081934

[32] Matreyek K. A. Multiplex assessment of protein variant abundance by massively parallel sequencing. Nat Genet 50, 874–882, doi:10.1038/s41588-018-0122-z (2018). 29785012 PMC5980760

[33] Wan A., Place E., Pierce E. A. & Comander J. Characterizing variants of unknown significance in rhodopsin: a functional genomics approach. Human mutation 40, 1127–1144 (2019). 30977563 10.1002/humu.23762 PMC7027811

[34] Verhoef L. G., Mattioli M., Ricci F., Li Y. C. & Wade M. Multiplex detection of protein-protein interactions using a next generation luciferase reporter. Biochim Biophys Acta 1863, 284–292, doi:10.1016/j.bbamcr.2015.11.031 (2016). 26646257

[35] Jones E. M. Structural and functional characterization of G protein-coupled receptors with deep mutational scanning. Elife 9, doi:10.7554/eLife.54895 (2020). PMC7707821 33084570

[36] Komarova E. S., Dontsova O. A., Pyshnyi D. V., Kabilov M. R. & Sergiev P. V. Flow-Seq Method: Features and Application in Bacterial Translation Studies. Acta Naturae 14, 20–37, doi:10.32607/actanaturae.11820 (2022). 36694903 PMC9844084

[37] Schwinn M. K. CRISPR-mediated tagging of endogenous proteins with a luminescent peptide. ACS chemical biology 13, 467–474 (2018). 28892606 10.1021/acschembio.7b00549

[38] Kamiyama D. Versatile protein tagging in cells with split fluorescent protein. Nature communications 7, 11046 (2016). 10.1038/ncomms11046 PMC4802074 26988139

[39] Liu X., Tao T., Zhao L., Li G. & Yang L. Molecular diagnosis based on comprehensive genetic testing in 800 Chinese families with non-syndromic inherited retinal dystrophies. Clin Exp Ophthalmol 49, 46–59, doi:10.1111/ceo.13875 (2021). 33090715

[40] Shi J. Clinical Features and Natural History in a Cohort of Chinese Patients with RPE65-Associated Inherited Retinal Dystrophy. J Clin Med 10, doi:10.3390/jcm10225229 (2021). PMC8625455 34830511

[41] Hanna R. E. Massively parallel assessment of human variants with base editor screens. Cell 184, 1064–1080. e1020 (2021). 33606977 10.1016/j.cell.2021.01.012

[42] Khanna A. Bam-readcount - rapid generation of basepair-resolution sequence metrics. ArXiv (2021). 10.21105/joss.03722 PMC13367885 42453900

[43] Yue P., Li Z. & Moult J. Loss of protein structure stability as a major causative factor in monogenic disease. Journal of molecular biology 353, 459–473 (2005). 16169011 10.1016/j.jmb.2005.08.020

[44] Preising M., Paunescu K., Friedburg C. & Lorenz B. Genetic and clinical heterogeneity in LCA patients: The end of uniformity. Der Ophthalmologe 104, 490–498 (2007). 17525851 10.1007/s00347-007-1533-x

[45] Jin M. Functional rescue of retinal degeneration-associated mutant RPE65 proteins. (Springer, 2016). 10.1007/978-3-319-17121-0_70 PMC5623592 26427455

[46] Choi E. H. Insights into the pathogenesis of dominant retinitis pigmentosa associated with a D477G mutation in RPE65. Human Molecular Genetics 27, 2225–2243 (2018). 29659842 10.1093/hmg/ddy128 PMC6005012

[47] Feathers K. L. Gene Supplementation in Mice Heterozygous for the D477G RPE65 Variant Implicated in Autosomal Dominant Retinitis Pigmentosa. Hum Gene Ther 34, 639–648, doi:10.1089/hum.2022.240 (2023). 37014074 PMC10354729

[48] Wu W., Takahashi Y., Ma X., Moiseyev G. & Ma J. X. Environmental Light Has an Essential Effect on the Disease Expression in a Dominant RPE65 Mutation. Adv Exp Med Biol 1415, 415–419, doi:10.1007/978-3-031-27681-1_61 (2023). 37440066

[49] Bereta G. Impact of retinal disease-associated RPE65 mutations on retinoid isomerization. Biochemistry 47, 9856–9865 (2008). 18722466 10.1021/bi800905v PMC2610467

[50] Jin M., Li S., Moghrabi W. N., Sun H. & Travis G. H. Rpe65 is the retinoid isomerase in bovine retinal pigment epithelium. Cell 122, 449–459 (2005). 16096063 10.1016/j.cell.2005.06.042 PMC2748856

[51] Li Y. Mouse model of human RPE65 P25L hypomorph resembles wild type under normal light rearing but is fully resistant to acute light damage. Human molecular genetics 24, 4417–4428 (2015). 25972377 10.1093/hmg/ddv178 PMC4492402

[52] Samardzija M. R91W mutation in Rpe65 leads to milder early-onset retinal dystrophy due to the generation of low levels of 11-cis-retinal. Human molecular genetics 17, 281–292 (2008). 17933883 10.1093/hmg/ddm304

[53] Takahashi Y., Chen Y., Moiseyev G. & Ma J.-x. Two point mutations of RPE65 from patients with retinal dystrophies decrease the stability of RPE65 protein and abolish its isomerohydrolase activity. Journal of Biological Chemistry 281, 21820–21826 (2006). 16754667 10.1074/jbc.M603725200

[54] Uppal S., Liu T., Poliakov E., Gentleman S. & Redmond T. M. The dual roles of RPE65 S-palmitoylation in membrane association and visual cycle function. Scientific Reports 9, 5218 (2019). 30914787 10.1038/s41598-019-41501-w PMC6435699

[55] Uppal S. An inducible amphipathic α-helix mediates subcellular targeting and membrane binding of RPE65. Life Science Alliance 6 (2023). 10.26508/lsa.202201546 PMC9585964 36265895

[56] Starita L. M. Massively parallel functional analysis of BRCA1 RING domain variants. Genetics 200, 413–422 (2015). 25823446 10.1534/genetics.115.175802 PMC4492368

[57] Tavtigian S. V., Harrison S. M., Boucher K. M. & Biesecker L. G. Fitting a naturally scaled point system to the ACMG/AMP variant classification guidelines. Human mutation 41, 1734–1737 (2020). 32720330 10.1002/humu.24088 PMC8011844

[58] Brnich S. E. Recommendations for application of the functional evidence PS3/BS3 criterion using the ACMG/AMP sequence variant interpretation framework. Genome Med 12, 3, doi:10.1186/s13073-019-0690-2 (2019). 31892348 PMC6938631

